# Construction of Porphyrin-Based Bimetallic Nanomaterials with Photocatalytic Properties

**DOI:** 10.3390/molecules29030708

**Published:** 2024-02-03

**Authors:** Zhiqiang Ji, Mengnan Yuan, Zhaoqin He, Hao Wei, Xuemin Wang, Jianxin Song, Lisha Jiang

**Affiliations:** 1 School of Civil Engineering, Yantai University, Yantai 264005, China; jzq@ytu.edu.cn; 2 School of Environmental and Materials Engineering, Yantai University, Yantai 264005, China; hezhaoqin2021@163.com (Z.H.); weixiaohao2021@163.com (H.W.); wxm3053@163.com (X.W.); sjx199908@163.com (J.S.)

**Keywords:** nanosheets, bimetallic, porphyrin, photocatalytic, singlet oxygen

## Abstract

The efficient synthesis of nanosheets containing two metal ions is currently a formidable challenge. Here, we attempted to dope lanthanide-based bimetals into porphyrin-based metal-organic skeleton materials (MOFs) by microwave-assisted heating. The results of the EDX, ICP, and XPS tests show that we have successfully synthesized porphyrin-based lanthanide bimetallic nanosheets (Tb-Eu-TCPP) using a household microwave oven. In addition, it is tested and experimentally evident that these nanosheets have a thinner thickness, a larger BET surface area, and higher photogenerated carrier separation efficiency than bulk porphyrin-based bimetallic materials, thus exhibiting enhanced photocatalytic activity and n-type semiconductor properties. Furthermore, the prepared Tb-Eu-TCPP nanomaterials are more efficient in generating single-linear state oxygen under visible light irradiation compared to pristine monometallic nanosheets due to the generation of bimetallic nodes. The significant increase in catalytic activity is attributed to the improved separation and transfer efficiency of photogenerated carriers. This study not only deepens our understanding of lanthanide bimetallic nanosheet materials but also introduces an innovative approach to improve the photocatalytic performance of MOFs.

## 1. Introduction

Metal–organic frameworks (MOFs) [1], also referred to as coordination polymers, are hybrid materials featuring intramolecular pores formed through the self-assembly of organic ligands and metal ions or clusters via coordination bonds [2]. These MOFs exhibit remarkable potential as multifunctional materials, owing to their high porosity and extensive specific surface area, and have been extensively explored for applications in gas storage and separation [3], sensing [4], catalysis [5], and biomedicine [6]. Lanthanide metal–organic framework materials are an important branch of MOFs; they are characterized by their sharp emissions, large choke offset, and relatively long luminescence lifetime [7,8]. However, these unique advantages also bring limitations to photocatalysis. When a lanthanide metal–organic skeleton is irradiated by light and enters the excited state, electrons absorb energy and move out of the orbitals, leaving behind holes. Due to a certain coordination pattern between the lanthanide metal and the organic ligand, the photogenerated electrons and holes recombine rapidly, which limits its photocatalytic performance [9,10]. Therefore, the choice of organic ligands is crucial in the synthesis of lanthanide metal–organic framework materials. Porphyrin [11] is an excellent organic ligand with a unique macrocyclic structure and efficient visible light-trapping ability. In recent years, there has been a strong level of interest in utilizing porphyrin-based MOF photocatalysts for various applications, such as photocatalytic hydrogen evolution [12,13], the photocatalytic reduction of carbon dioxide [14,15,16], and photocatalytic organic reactions [17]. For example, Qi et al. [18] developed Co-Nd and Ni-Nd PMOF materials for the effective photocatalytic oxidation of benzyl alcohol and benzylamine under moderate air conditions. ARR et al. [19] reviewed the use of porphyrin-based or porphyrin-containing MOFs and COFs, including nanomaterials as heterogeneous single-linear state oxygen photosensitizers, for antimicrobial applications in recent times.

Despite the promise that MOFs hold in terms of structure tuning and composite materials for property enhancement, there remains a need for significant improvements in their performance. On the other hand, since the discovery of materials like graphene [20,21,22], it has become evident that 2D materials, characterized by their extensive surface areas and relatively high surface energies, possess unique physicochemical properties unrivaled by bulk crystalline materials. Two-dimensional metal backbone materials [23,24], a recent addition to the realm of 2D materials, have captured significant attention due to their unique attributes. Compared to the typical three-dimensional MOF crystals, 2D MOFs offer wider planar dimensions and ultra-small thicknesses, granting 2D materials an expanded specific surface area and more exposed active sites, particularly beneficial for catalytic and sensing applications [25,26,27]. Up until now, 2D MOF-based catalysts have attracted widespread research interest in electrocatalysis, photocatalysis, and thermocatalysis.

According to the relevant literature reports, we know that when certain lanthanide ions are doped in the catalysts, they can realize the up-conversion from visible to ultraviolet light of the substances [28], the extension of the response region [29], as well as the improvement of the separation efficiency of the photogenerated electron–hole pairs [30,31], which can improve the catalytic activity of the catalysts. Inspired by ultra-thin nanomaterials [32,33] and the MOFs of bimetallic porphyrins [34], we attempted to incorporate two lanthanide elements into porphyrin nanosheets to investigate their effects on photocatalytic performance. In this study, porphyrin-based nanosheet materials containing two lanthanide metal ions were synthesized using microwave-assisted heating, with acetic acid serving as a moderator. The prepared bimetallic nanosheets exhibit a large BET specific surface area and unique metal sites. Notably, the synthesized lanthanide bimetallic porphyrin nanomaterials demonstrated superior photocatalytic properties compared to both mono-metallic and bulk bimetallic materials. The results indicate that incorporating multi-component metal ions in nanomaterials can effectively enhance the catalytic efficiency of nanomaterial catalysts, offering a promising avenue for improving the photocatalytic performance of nanomaterials.

## 2. Results and Discussion

**Figure 1:**
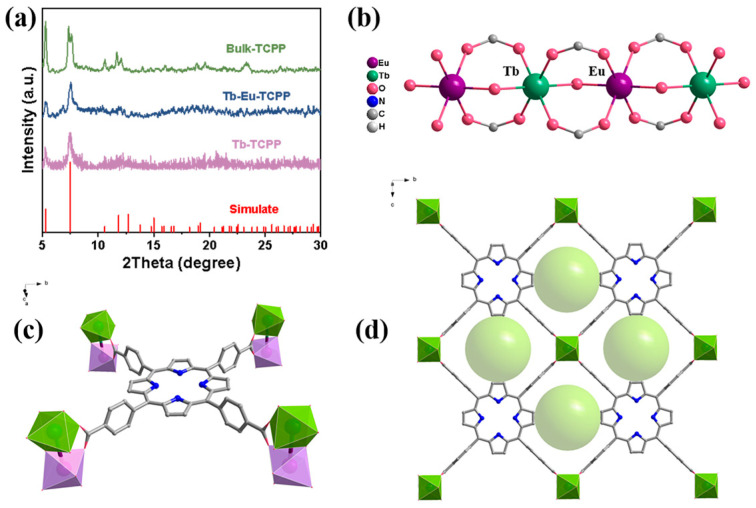
(**a**) The XRD spectra of Bulk-TCPP, Tb-Eu-TCOPP, and Tb-TCPP, and the simulate of Tb-TCPP; (**b**) the possible one-dimensional direct connections of Tb and Eu to oxygen; (**c**) the coordination environments of Tb (III) ions, Eu (III) ions, and the TCPP ligand; and (**d**) views of the 1D channels (highlighted by green balls).

The crystal structures of the synthesized samples were measured by powder XRD spectroscopy. As shown in Figure 1a, all the samples showed better crystallinity and similar diffraction peaks in the 2θ (5°–30°) range. Furthermore, comparing them with the XRD of the known crystal Tb-TCPP (CCDC: 1899682), it was found that all the materials have similar diffraction peaks, which suggests that these materials have a similar structure to Tb-TCPP. Since Tb and Eu belong to the same lanthanide metal and have similar chemical properties, they have similar coordination environments during the synthesis of crystals, and during the synthesis process of doping two metals, Tb and Eu may be coordinated at the same position [34,35,36]. In addition, the concentrations of Tb and Eu were obtained through ICP testing. The ratio of the two lanthanide elements was calculated to be Tb:Eu = 1.1:1 (Table S1). This result is close to the ratio of the preparation process (Tb:Eu = 1:1). Therefore, when studying the structure, a 1:1 ratio of Tb and Eu was adopted. On this basis, the possible crystal structure formed by Tb-Eu-TCPP is simulated by the known crystal structure [37], as shown in Figure 1. The Tb/Eu atom adopts a pseudo-octahedral coordination with the oxygen atoms of four bridging carboxyl groups and two μ_2_-OH groups of four different TCPP linkers to form a 1D chain along the a-axis direction (Figure 1b). The porphyrin centers are metal-free, nearly planar, and have no apparent out-of-plane deformation (Figure 1c). A one-dimensional channel constructed from each carboxylate in TCPP and two Tb/Eu atoms from the 1D chain is shown in Figure 1d (the channel is highlighted with a green ball).

**Figure 2:**
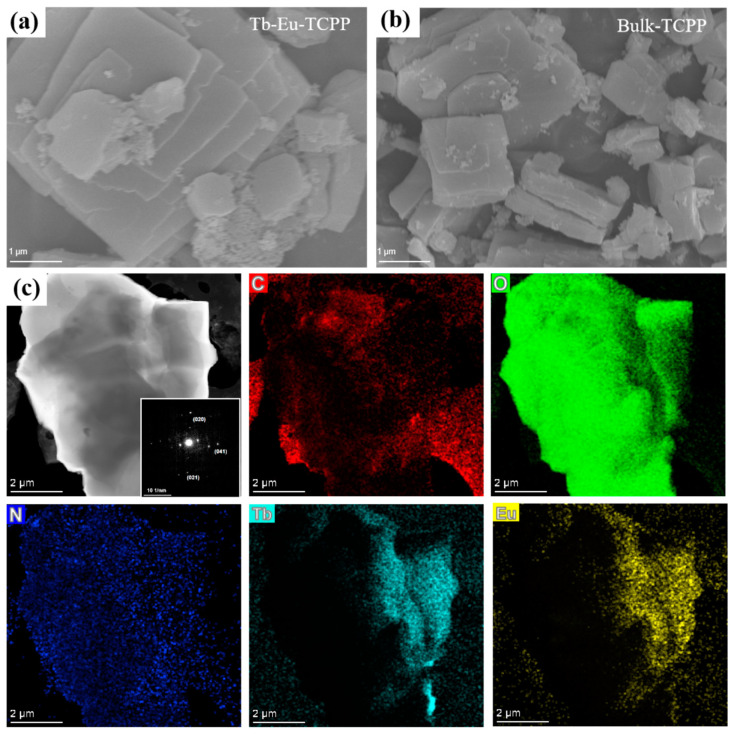
(**a**) SEM images of Tb-Eu-TCPP nanosheets; (**b**) SEM images of Bulk-TCPP; and (**c**) TEM images and relevant elemental mapping of Tb-Eu-TCPP. Inset: SAED pattern of Tb-Eu-TCPP nanosheets.

Subsequently, the morphology and structure of Tb-Eu-TCPP were measured by scanning electron microscopy (SEM) and transmission electron microscopy (TEM). The bright spots were clearly presented in a selected area electron diffraction (SAED) pattern, which were attributed to the (020), (021), and (041) planes of the Tb-Eu-TCPP nanosheets, confirming the crystal structure (inset in Figure 2a). This result is in excellent agreement with the three typical peaks (020), (021), and (041) in the XRD and simulated pattern of Tb-Eu-TCPP and Bulk-TCPP (Figure 1a). The contents shown in Figure 2a,b are the SEM images of Tb-Eu-TCPP and Bulk-TCPP. Unlike the hydrothermal method [38,39], microwave radiation induces localized superheating. The selective adsorption of solvent molecules or moderators on the crystal surface under microwave radiation plays a crucial role in the anisotropic growth of 2D nanosheets, leading to the formation of these distinct 2D nanosheets [40,41,42]. Simultaneously, acetic acid was used as a moderating conditioner, which was effective in inhibiting the deprotonation of carboxyl groups within the ligand [43,44]. Therefore, an increase in the amount of acetic acid in the hydrothermal process during the reaction results in a reduced number of nucleation events and increased inhibition of lateral growth in the nanosheets, leading to the thickening of the prepared nanosheets and the formation of a bulkier structure. TEM characterization further revealed the morphology of Tb-Eu-TCPP, with C, O, N, Tb, and Eu elements evenly distributed on the octahedron (Figure 2c). In addition, the results of elemental analysis testing further show that the material contains C, H, and N elements (Table S2). According to the test results of EDX (Figure S3) and ICP (Table S1), both Tb and Eu lanthanide metals were present in Tb-Eu-TCPP. The results of TEM and ICP indicate that the bimetallic Tb-Eu-TCPP was successfully synthesized.

**Figure 3:**
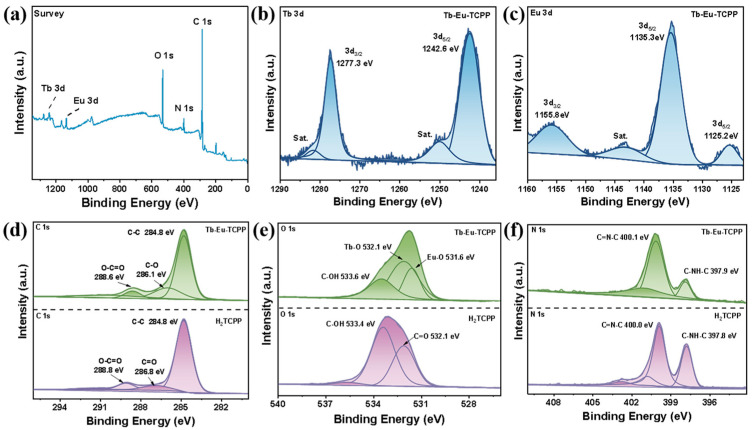
The XPS spectra of the Tb-Eu-TCPP nanosheets and H_2_TCPP: survey spectrum of Tb-Eu-TCPP (**a**) and high-resolution spectra of Tb 3d (**b**), Eu 3d (**c**), C 1s (**d**), O 1s (**e**), and N 1s (**f**).

X-ray photoelectron spectroscopy (XPS) was utilized to explore the chemical state and bonding configuration of H_2_TCPP and Tb-Eu-TCPP. Figure 3a shows the presence of N, O, and C elements in H_2_TCPP and Tb, Eu, N, O, and C elements in Tb-Eu-TCPP. The binding energy data have been corrected for C-C at 284.8 eV (Figure 3d) [45]. The high-resolution spectrum of Tb 3d was deconvoluted into four peaks (Figure 3b). The peaks at 1281.6 eV and 1249.9 eV were satellite signals (abbreviated as “Sat.”). The two peaks at 1242.6 eV and 1277.3 eV were attributed to Tb 3d_5/2_ and Tb 3d_3/2_, respectively [35]. The high-resolution spectrum of Eu 3d was deconvoluted into four peaks (Figure 3c). The peak at 1143.1 eV was a satellite signal. The peak at 1135.3 eV was attributed to Eu^3+^. Two smaller Eu 3d_5/2_ and Eu 3d_3/2_ peaks were observed at 1125.2 eV and 1155.8 eV, respectively, attributed to the Eu^2+^ oxidation state [46,47]. The corresponding O 1s XPS profiles were deconvoluted into three peaks of 531.6 eV, 532.1 eV, and 533.6 eV, among which the peak at 533.6 eV was associated with C-OH bonds, the peak at 532.6 eV was ascribed to Tb-O bonds, and the peak located at 531.6 eV came from Eu-O bonds [48,49]. The above results all indicated the successful preparation of Tb-Eu-TCPP. Figure 3f displays the N 1s energy spectrum of the Tb-Eu-TCPP nanosheets alongside the H_2_TCPP ligand. Among the characteristic energy spectrum peaks of the H_2_TCPP ligand, the peaks at 400.0 and 397.8 eV correspond to the C=N-C and C-NH-C groups, respectively [50,51]. Notably, the N 1s energy spectrum of the Tb-Eu-TCPP nanosheets did not exhibit significant changes when compared to the characteristic N 1s peaks of H_2_TCPP. In the characteristic energy spectrum peaks of the H_2_TCPP ligand, the peaks corresponding to the C=N-C and C-NH-C groups are 400.1 and 397.9 eV, respectively. This indicates that the porphyrin ring is not coordinated with Tb and Eu. In addition, the results of XPS demonstrate that during the formation of Tb-Eu-TCPP, Tb/Eu only coordinates with the O in the carboxyl group to form a Tb/Eu-O bond. Meanwhile, Tb/Eu does not bond with the N in the center of the porphyrin ring. Therefore, the results of XPS further demonstrate that in the crystal structure of Tb-Eu-TCPP, Tb/Eu forms a strong coordination with the carboxyl group in the porphyrin, forming a one-dimensional chain structure.

**Figure 4:**
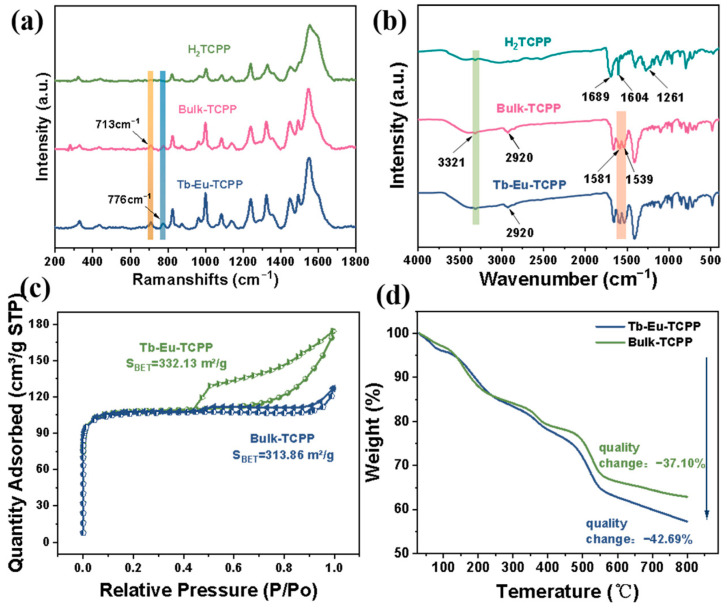
(**a**) Raman spectra of H_2_TCPP with different states of Tb-Eu-TCPP nanosheets; (**b**) IR spectra of H_2_TCPP with different states of Tb-Eu-TCPP nanosheets; (**c**) BET and N_2_ adsorption–desorption isothermal curves of Tb-Eu-TCPP nanosheets and Bulk-TCPP; and (**d**) TGA of Tb-Eu-TCPP nanosheets and Bulk-TCPP.

Moreover, the Raman spectra of Tb-Eu-TCPP nanosheets and Bulk-TCPP show typical characteristics of H_2_TCPP except for the peaks at 713 cm^−1^ and 776 cm^−1^, which are assigned to Tb-O and Eu-O, respectively (Figure 4a) [52,53]. Figure 4b shows the FTIR spectroscopy results acquired for the aforementioned materials. By analyzing the results obtained from Tb-Eu-TCPP, it can be observed that there are significant peaks at 3321 and 2920 cm^−1^ in the range of 4000–2000 cm^−1^. Among these peaks, the peak at 3321 cm^−1^ corresponds to the OH-stretching vibration [54,55]. This is consistent with the peak position in the ligand, indicating that there may be less TCPP in the substance in the free state. Notably, the band at 2920 cm^−1^ was attributed to intergranular water and the intracavity physical absorption of water [56,57]. In the range of 500–2000 cm^−1^, the shoulder peaks at 1689 cm^−1^ and 1604 cm^−1^ assigned to the asymmetric stretching vibration of C=O in H_2_TCPP were shifted to 1581 cm^−1^ and 1539 cm^−1^, respectively, and significantly decreased in their intensity for the Tb-Eu-TCPP nanosheets. The disappearance of the stretching vibration of C-O at 1261 cm^−1^ can be also observed in the spectra of Bulk-TCPP and Tb-Eu-TCPP nanosheets [7,55,58]. All these results demonstrate that the Tb-Eu-TCPP nanosheets are constructed by the strong coordination of Tb^3+^ (Eu^3+^) with the carboxyl groups in H_2_TCPP.

The relative changes in the specific surface area of the Tb-Eu-TCPP nanosheets and Bulk-TCPP were investigated through N_2_ adsorption–desorption isotherm experiments. As illustrated in Figure 4c, the nanosheet material exhibited a significantly higher specific surface area compared to the bulk material. Specifically, the BET specific surface area of the Tb-Eu-TCPP nanosheets was 332.13 m^2^/g, whereas that of Bulk-TCPP was 313.86 m^2^/g. This exceptional specific surface area suggests that these materials have excellent catalytic performance potential [59,60,61]. The pore size distribution curves (Figure S4) indicate that the Tb-Eu-TCPP nanosheets have similar micropores as Bulk-TCPP. The thermal stability of the Tb-Eu-TCPP nanosheets and Bulk-TCPP was assessed using thermogravimetric analysis (TGA). As illustrated in Figure 4d, the TGA curves of the Tb-Eu-TCPP nanosheets and Bulk-TCPP exhibit a high degree of similarity. Within the temperature range of 30–350 °C, the weight loss is approximately 20%. Subsequently, a sharp decline in weight is observed at 500 °C, indicating the onset of nanosheet backbone collapse. These results indicate that both Tb-Eu-TCPP nanosheets and Bulk-TCPP possess high thermal stability, maintaining the integrity of the backbone under nitrogen, and do not undergo thermal decomposition up to 500 °C.

Porphyrin-based MOFs are well-established for their applications in singlet oxygen (^1^O_2_) generation within the realms of photocatalysis and photodynamic therapy [62,63,64]. To determine the production of ^1^O_2_ species in the photocatalytic process, 9,10-dibenzanthracene (DPA) was used as a probe for ^1^O_2_ to assess the ability of Tb-Eu-TCPP nanosheets to generate ^1^O_2_ under visible light irradiation. DPA functions as an ^1^O_2_-trapping agent, with the concentration of DPA decreasing as it traps ^1^O_2_.

**Figure 5:**
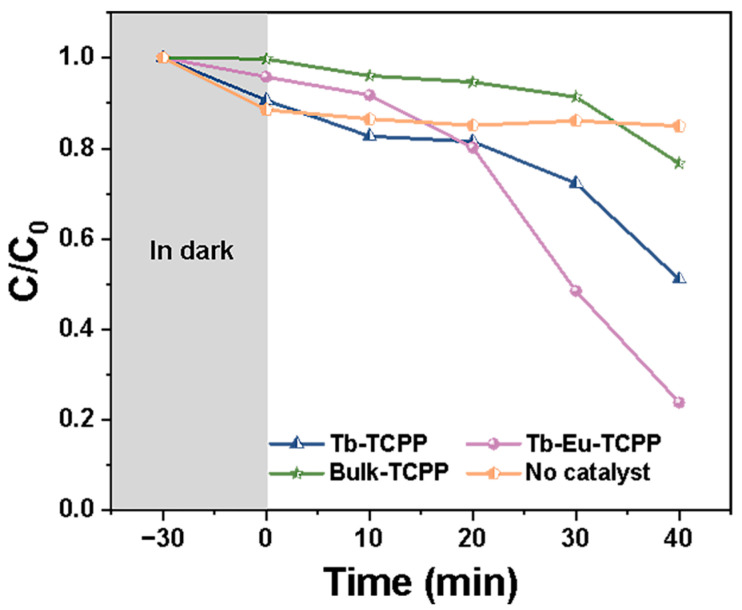
Photocatalytic performance of DPA with time in different solution systems.

The concentration of DPA was monitored by measuring its absorbance at 374 nm using a UV–visible spectrophotometer. Figure 5 illustrates the concentration curves of DPA over time in different solution systems. Subsequently, Figure S5a–d display the absorption spectra of DPA over time for the Tb-TCPP nanosheets, Tb-Eu-TCPP nanosheets, Bulk-TCPP, and the system without added catalyst, respectively. The analysis of the data in Figure 5 leads to the following conclusions: In the presence of the Tb-Eu-TCPP nanosheets, the concentration of DPA consistently decreases over time, indicating the consumption of DPA during the process and providing evidence for the generation of singlet oxygen (^1^O_2_). Furthermore, compared to Tb-TCPP, Bulk-TCPP systems, or systems without added catalyst, there is no significant decrease in the concentration of DPA. This result emphasizes the enhanced photocatalytic activity of thinner Tb-Eu-TCPP nanosheets. Additionally, the results demonstrate that bimetallic nanosheets exhibit better catalytic effects compared to monometallic nanosheets.

**Figure 6:**
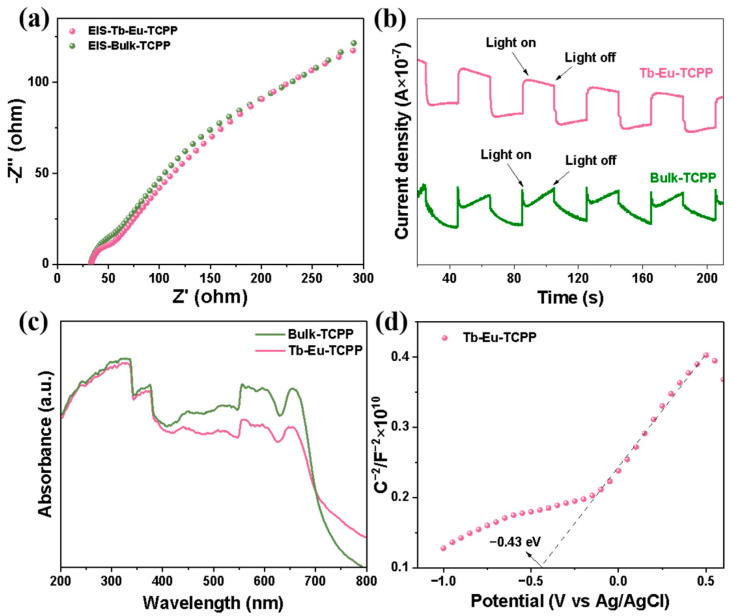
(**a**) EIS of Tb-Eu-TCPP nanosheets and Bulk-TCPP; (**b**) photocurrent response curves of Tb-Eu-TCPP nanosheets and Bulk-TCPP; (**c**) UV–Vis absorption spectra of Tb-Eu-TCPP nanosheets and Bulk-TCPP; and (**d**) Mott–Schottky curves of Tb-Eu-TCPP nanosheets and Bulk-TCPP samples.

In order to gain a deeper understanding of the mechanism of photocatalytic activity depending on the thickness and metal nodes, photocurrent tests were conducted to evaluate their charge separation efficiency. In Figure 6a, the plots display electrical impedance data for nanosheets with varying thicknesses. The test results reveal that the radius of the arc in the EIS plots for the Tb-Eu-TCPP nanosheets is smaller than that of Bulk-TCPP. This smaller arc radius suggests that the separation efficiency of photogenerated electron pairs is higher in the Tb-Eu-TCPP nanosheets, indicating an enhanced photocatalytic effect. Figure 6b presents photocurrent data for nanosheets with different thicknesses. Based on the experimental data, it is evident that the photocurrent density of Tb-Eu-TCPP nanosheets exceeds that of Bulk-TCPP, signifying that thinner nanosheets exhibit a higher separation efficiency of electrons and holes. This phenomenon suggests the potential for increased production of ·O_2_^−^ during the experimental process. Taking into account the previously conducted characterization, it is shown that thinner Tb-Eu-TCPP nanosheets have a greater BET specific surface area, light-trapping capacity, carrier density, and hole separation efficiency, all of which boost photocatalytic activity [65,66,67]. Additionally, the UV–Vis diffuse reflectance spectra reveal a similar band gap between the Tb-Eu-TCPP nanosheets and Bulk-TCPP, as depicted in Figure 6c. This suggests that the electron-leaping ability of both materials is comparable.

To determine the conduction band bottom potential (E_CB_) of the prepared Tb-Eu-TCPP, Mott–Schottky analysis was employed [68,69,70]. The results in Figure 6a,d reveal that Tb-Eu-TCPP exhibits n-type semiconductor behavior with a flat-band potential E_fb_= −0.43 V (Ag/AgCl). For n-type semiconductors, E_fb_ is typically 0.1 or 0.2 V more positive than the conduction band bottom potential (E_CB_). Consequently, the E_CB_ of Tb-Eu-TCPP is −0.53 V (Ag/AgCl). By applying the conversion equation for the standard hydrogen potential (NHE) and Ag/AgCl potential (E_NHE_ = E_Ag/AgCl_ + 0.197 V), the E_NHE_ of Tb-Eu-TCPP is calculated to be −0.34 V (NHE). From Figure S6, the band gap (E_g_) of the Tb-Eu-TCPP photocatalyst is 2.8 eV with bimetallic 2D properties. The valence band top potential (E_VB_) of Tb-Eu-TCPP is calculated to be 2.27 V (NHE) using the equation E_VB_ = E_NHE_ + E_g_. This establishes a high oxidation–reduction potential for electron/hole pairs, allowing them to engage with dissolved oxygen and generate a substantial quantity of reactive oxygen species [71], notably ^1^O_2_.

**Figure 7:**
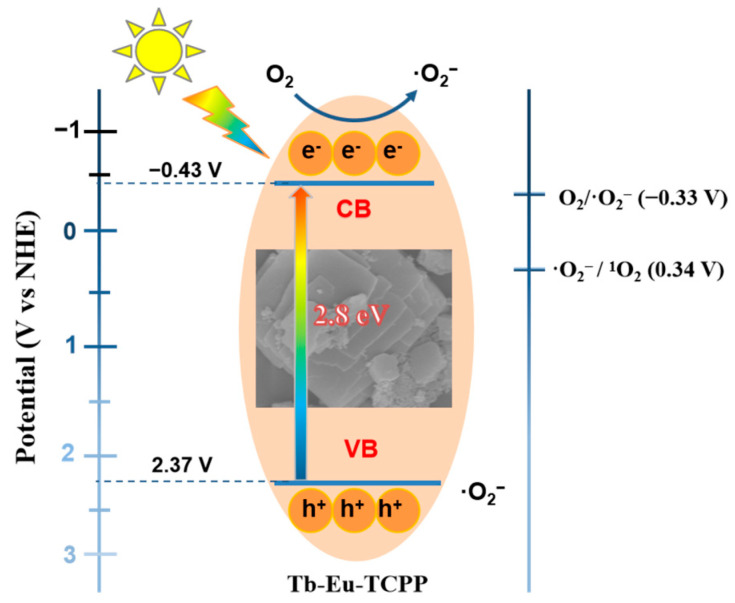
Mechanism diagram of ^1^O_2_ generation by Tb-Eu-TCPP photocatalysis.

In the system of bimetallic composites, the combined effect of the bimetals accelerates the charge transfer and inhibits electron and hole complexation, which improves the photocatalytic activity of this catalyst. Meanwhile, in the photocatalytic system of Tb-Eu-TCPP, light energy mainly drives the charge transfer, which leads to the generation of hydrated electrons upon photoexcitation. These hydrated electrons are subsequently trapped by oxygen to produce superoxide anion radicals (·O_2_^−^), and ·O_2_^−^ can further react with holes to produce ^1^O_2_ [72]. This principle is the same as outlined by Demyanenko et al. [73], who proposed an alternative mechanism of the photocatalytic generation of singlet oxygen through the photodetachment of an electron from the superoxide radical anion. In other words, once generated through the single-electron reduction of oxygen (Equation (1)), the superoxide radical anion absorbs light, giving rise to singlet oxygen and releasing an electron (Equation (2)), which, in turn, can recombine or reduce another oxygen molecule. The possible reactions are schematically shown in Figure 7.

(1) $$O^{2}+e^{-}\to \cdot O_{2}^{-}$$

(2) ·O2−+hv→O21+e−

## 3. Materials and Methods

### 3.1. Materials

Tb(NO_3_)_3_·6H_2_O, Eu(NO_3_)_3_·6H_2_O, meso-Tetra(4-carboxyphenyl) porphyrin (H_2_TCPP), 9,10-diphenylanthracene (DPA), acetonitrile (MeCN), acetic acid (HAc), and N-N dimethylformamide (DMF) were all employed in this study. All the reagents used in the experiments were of commercial grade and did not require additional purification. Unless specified otherwise, ultrapure water was used in the experiments.

### 3.2. Characterization and Instruments

The Midea brand home microwave oven M1-L213B (Midea, Shanghai, China) was used to prepare Tb-Eu-TCPP nanosheet samples. A JSM-7610F (JEOL Ltd., Tokyo, Japan) scanning electron microscope was used to characterize the morphology and size of the prepared samples. A transmission electron microscope was used for the morphological and selected area electron diffraction characterization of the samples with the FEI Tecnai G2 instrument model. An atomic force microscope (Dimension ICON) (Bruker Corporation, NASDAQ, Billerica, MA, United States) was used to characterize the thickness of the samples. A Smartlab 3 X-ray (JEOL Ltd., Tokyo, Japan) diffractometer was used to collect the powder diffraction data of the samples. Nitrogen adsorption–desorption isotherms of the samples were examined using an ASAP 2460 (Micromeritics, Norcross, Norcross, GA, United States) fully automated gas adsorption analyzer. Thermogravimetric analysis of the samples was carried out on an NETZSCH STA 2500 (Netzsch, Selb, Germany) thermogravimetric analyzer. The material was tested for Tb and Eu using an Agilent 7800 ICP-MS tool (Agilent, Santa Clara, CA, United States). The TEM and EDX of the material were tested using Talos F200X G2 (Thermo scientific, Waltham, MA, United States) to characterize the material’s morphology and elemental information. The elemental analysis of the materials was conducted using an Elementar Unicube (Elementar, Langenselbold, Germany). A Thermo ESCELAB 250 XI X-ray photoelectron spectrometer (Thermo scientific, Massachusetts, United States) was used to characterize the X-ray photoelectron spectra of the samples. A RENISHAW InVia Raman microscope (Renishaw, London, England) was used to test the Raman spectra of the samples. The Fourier transform infrared spectrometer FTIR Prestige-21 (Shimadzu, Kyoto, Japan) was used to characterize the infrared spectra of the samples. The UV–Vis absorption spectra and solid diffuse reflectance spectra of the samples were tested and recorded using a UV2600 spectrophotometer (Techcomp, Shanghai, China). A 300 W xenon lamp (CEL-HXF300, China Education Au-light Co., Ltd., Beijing, China) was used to provide the light source. All electrochemical measurements were performed on a CHI 760E (CH Instruments, Shanghai, China) electrochemical workstation.

### 3.3. Synthesis of Tb-Eu-TCPP Nanosheets

First, 0.045 mmol Tb(NO_3_)_3_·6H_2_O (20.4 mg), 0.045 mmol Eu(NO_3_)_3_·6H_2_O (20.1 mg), and 0.03 mmol H_2_TCPP (23.7 mg) were dissolved in 9 mL of DMF. Then, 100 μL of each of the 100 μL of the above-mixed solutions were taken into a 3-mL high-temperature-resistant glass vial. After that, 5 μL of a glacial acetic acid solution was added to it, which was labeled as Tb-Eu-TCPP. The mixed solution was diluted to 1 mL with DMF and then sealed tightly with the cap of the vial and placed in a household microwave oven at medium–low heat (its power was about 231 W) and microwaved for 10 min. After cooling to room temperature, the reaction was washed twice with DMF and anhydrous ethanol by centrifugation at 5000 rpm for 10 min. After washing, they were dried at room temperature. The specific schematic diagram of the synthesis process is shown in Figure S1.

### 3.4. Synthesis of Bulk-TCPP

Firstly, 0.019 mmol Tb(NO_3_)_3_·6H_2_O (8.5 mg) plus 0.013 mmol Eu(NO_3_)_3_·6H_2_O (8.6 mg) and 0.03 mmol H_2_TCPP (23.7 mg) were dissolved in 10 mL of DMF; then, 5 mL of the above-mixed was transferred into a PTFE-lined stainless steel autoclave, following which 400 μL of acetic acid was added, and it was placed in a 120 °C blast drying oven for 12 h. After being cooled down to room temperature, the Tb-Eu-TCPP block was washed by centrifugation with DMF and anhydrous ethanol two times. Centrifugation conditions: 5000 rpm for 10 min; after washing, it was dried at room temperature. The specific schematic diagram of the synthesis process is shown in Figure S2.

### 3.5. Monitoring of Singlet Oxygen Using DPA as a Probe

To validate the generation of singlet oxygen (^1^O_2_) under exposure to Tb-Eu-TCPP light, 9,10-diphenylanthracene (DPA) was employed as an ^1^O_2_-trapping agent. The experimental procedure was as follows: a solution of DPA (100 μg/mL) was prepared using an oxygen-saturated acetonitrile/water (4:1) solvent mixture. Additionally, a dispersion of Tb-Eu-TCPP nanosheets (10 μg/mL) was prepared. Subsequently, 10 mL of the DPA (100 μg/mL) solution and Tb-Eu-TCPP nanosheet (10 μg/mL) dispersion were mixed in a tube. The mixture was then exposed to a 300 W xenon lamp equipped with a UV cut-off filter, and samples were collected at different exposure times for testing UV–visible absorption spectra.

## 4. Conclusions

In conclusion, we have successfully synthesized nanosheets of porphyrin-based bimetallic compositions, Tb-Eu-TCPP, using a microwave-assisted method, as well as bulk porphyrin-based bimetallic materials, Bulk-TCPP, using a hydrothermal method. In this study, we succeeded in preparing ultrathin bimetallic nanosheets, and compared with the thicker bimetallic Bulk-TCPP, the prepared Tb-Eu-TCPP nanosheets have a thinner thickness, a larger BET surface area, higher photogenerated carrier separation efficiency, and better photocatalytic activity, thus exhibiting n-type semiconductor properties. This study marks a significant progress in the controlled synthesis of bimetallic-doped nanosheet materials and opens up new possibilities for synthesizing bimetallic nanosheet materials.

## Data Availability

Data are contained within the article and Supplementary Materials.

## Supplementary Materials

The following supporting information can be downloaded at: www.mdpi.com/article/10.3390/molecules29030708/s1, Figure S1: Tb-Eu-TCPP schematic diagram of nanosheet synthesis; Figure S2: Bulk-TCPP schematic diagram of nanosheet synthesis; Figure S3: EDX of Tb-Eu-MOF obtained by TEM; Figure S4: Pore size distribution curves corresponding to Tb-Eu-TCPP (a) as well as Bulk-TCPP (b); Figure S5: The absorption spectra of DPA over time for Tb-TCPP nanosheets (a), Tb-Eu-TCPP nanosheets (b), Bulk-TCPP (c), and the system without added catalyst (d), respectively; Figure S6: VB-XPS spectra of Tb-Eu-TCPP nanosheets in different states; Table S1: ICP MS of Tb-Eu-TCPP; Table S2: Elemental analytics of Tb-Eu-TCPP.
