# Supplementary material for: Construction of Porphyrin-Based Bimetallic Nanomaterials with Photocatalytic Properties
Source: Molecules. 2024 Feb 3;29(3):708. doi: 10.3390/molecules29030708 (PMC10856655; doi:10.3390/molecules29030708)
Supplement: Supplementary file 1 [file molecules-29-00708-s001.zip › molecules-2798610-supplementary.pdf]

Supporting information

Construction of porphyrin-based bimetallic nanomaterials with photocatalytic properties

Zhi-Qiang Ji [1,†], Meng-Nan Yuan [1,†,*], Zhao-Qin He [2], Hao Wei [2], Xue-Min Wang [2], Jian-Xin Song [2], and Li-Sha Jiang [2,*]

[1] School of Civil Engineering, Yantai University, Yantai 264005, China; jzq@ytu.edu.cn (Z.-Q.J.)
[2] School of Environmental and Materials Engineering, Yantai University, Yantai 264005, China; hezhaoqin2021@163.com (Z.-Q.H.); weixiaohao2021@163.com (H.W.); wxm3053@163.com (X.-M.W.); Sjx199908@163.com (J.-X.S.)
[†] These authors contributed equally to this work.
[*] Correspondence: ytuymn@163.com (M.-N.Y.); jianglisha0112@ytu.edu.cn (L.-S.J.)

Supplementary Figure S1

Tb-Eu-TCPP schematic diagram of nanosheet synthesis

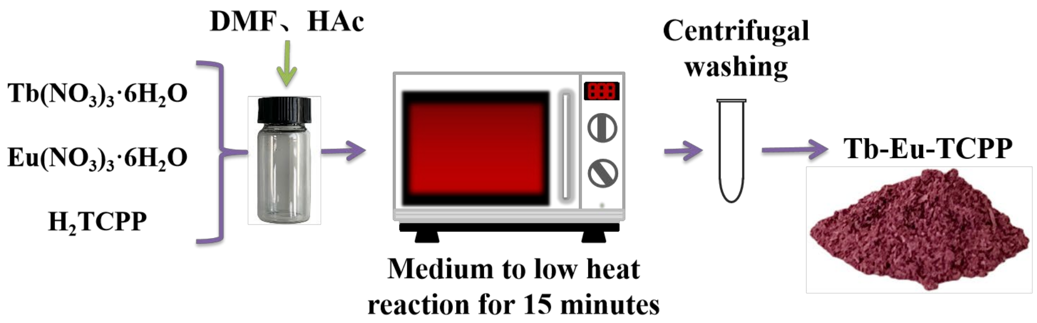

Supplementary Figure S2

Bulk-TCPP schematic diagram of nanosheet synthesis

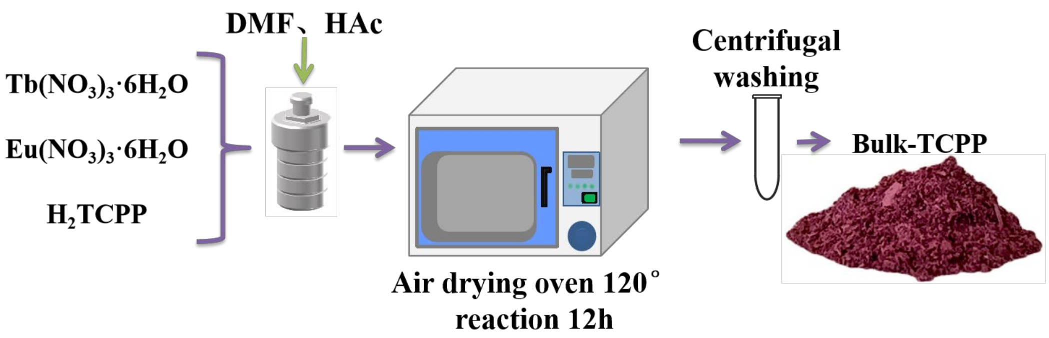

Supplementary Figure S3

EDX of Tb-Eu-MOF obtained by TEM

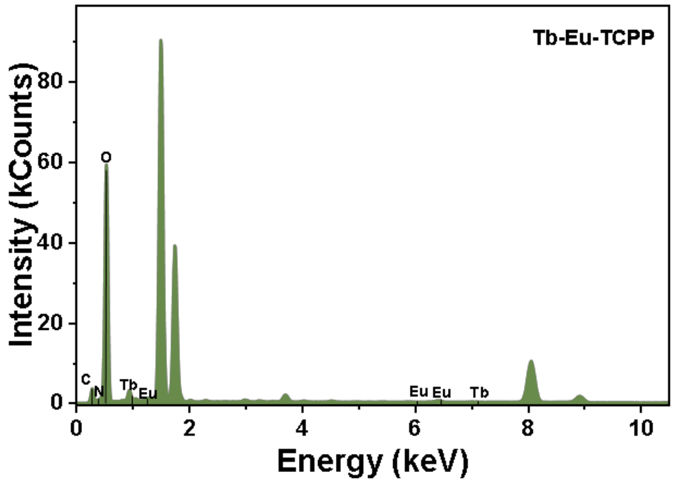

Supplementary Figure S4

Pore size distribution curves corresponding to Tb-Eu-TCPP (a) as well as Bulk-TCPP (b)

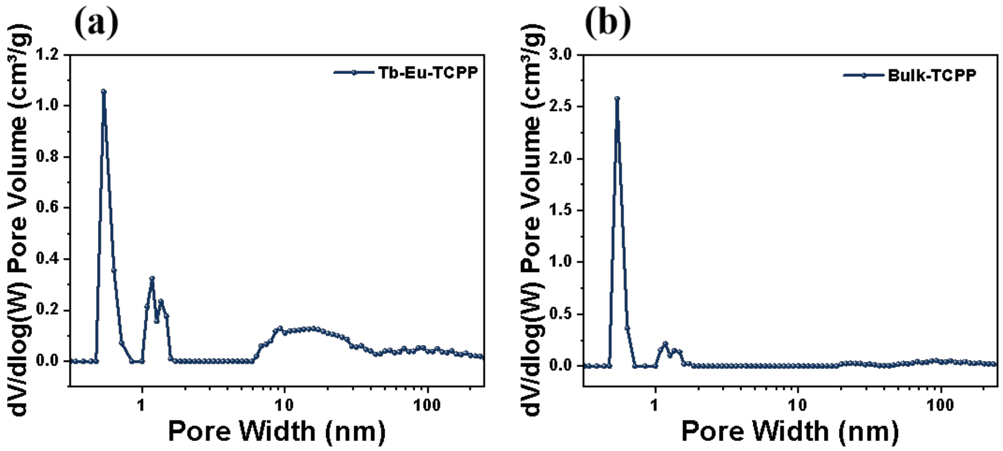

Supplementary Figure S5

The absorption spectra of DPA over time for Tb-TCPP nanosheets (a), Tb-Eu-TCPP nanosheets (b), Bulk-TCPP (c), and the system without added catalyst (d), respectively

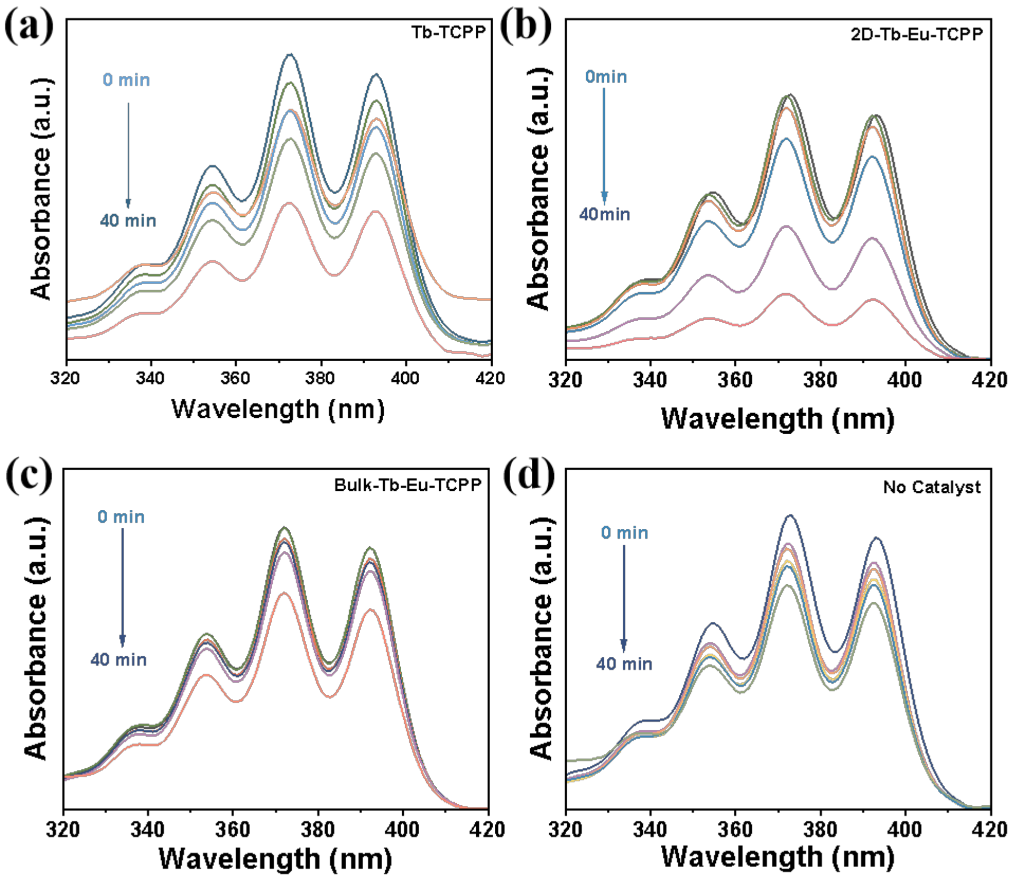

Supplementary Figure S6

VB-XPS spectra of Tb-Eu-TCPP nanosheets in different states

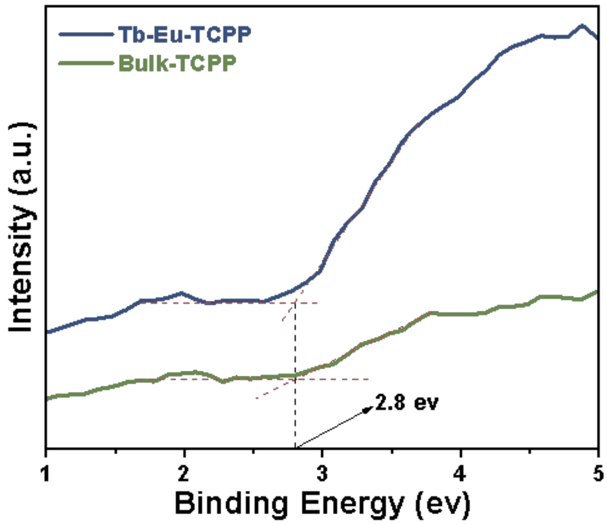

Supplementary Table S1

ICP MS of Tb-Eu-TCPP

ICP MS of Tb-Eu-TCPP				
**Element**	**Quality**	**Element test concentration (mg/L)**	**Element content (mg/Kg)**	**Element content (%)**
**Tb**	**1.2 mg**	**22.452×10^{-3}**	**94166.67**	**9.42**
**Eu**	**1.2 mg**	**19.316×10^{-3}**	**80833.33**	**8.08**

Supplementary Table S2

Elemental analytics of Tb-Eu-TCPP

Elemental analytics of Tb-Eu-TCPP			
**Elemental**	**N**	**C**	**H**
**Proportion (%)**	**5.49**	**48.64**	**3.17**
